# The impact of the national bowel screening program in the Netherlands on detection and treatment of endoscopically unresectable benign polyps

**DOI:** 10.1007/s10151-017-1705-x

**Published:** 2017-11-17

**Authors:** C. C. M. Marres, C. J. Buskens, E. Schriever, P. C. M. Verbeek, M. W. Mundt, W. A. Bemelman, A. W. H. van de Ven

**Affiliations:** 1 Department of Surgery, Flevoziekenhuis, Hospitaalweg 1, 1315 RA Almere, The Netherlands; 2 Department of Surgery, Academisch Medisch Centrum, Amsterdam, The Netherlands; 3 Department of Gastroenterology, Flevoziekenhuis, Almere, The Netherlands

**Keywords:** Colonic polyps, Colorectal surgery, Colorectal resection, Bowel screening program

## Abstract

**Background:**

In January 2014, a national bowel cancer screening program started in the Netherlands. The program is being implemented in phases until 2019. Due to this program, an increase in patients referred for a colorectal resection for benign, but endoscopically unresectable polyps, is expected. So far, most resections are performed according to oncological principles despite no pre-operative histological diagnosis of malignancy. The aim of this study was to analyze the increase in referred patients during the first year of the screening program and to compare pathological results and clinical outcome of resections of patients undergoing resection for benign polyps before and after implementation of screening.

**Methods:**

Patients referred for colorectal resection without biopsy-proven cancer between January 2009 and January December 2014 were identified from a prospectively maintained database. Patients with endoscopically macroscopic features of carcinoma were excluded.

**Results:**

Seventy-six patients were included. Forty-seven patients (61.8%) were operated on in the 5 years prior to implementation of the screening program, and 29 patients (38.2%) were operated during the first year of implementation of the screening program. The overall malignancy rate before the introduction of the program was 14.1 and 6.6% after it had started (*p* = .469). All resections were performed laparoscopically; the conversion rate was 3.9% (*n* = 3). The overall mortality rate was 2.7% (*n* = 2), major complications (Clavien–Dindo > 3b) occurred in 11.8% (*n* = 9) of patients. The anastomotic leakage rate was 3.9% (*n* = 3).

**Conclusions:**

The number of patients referred for benign polyps tripled after introduction of the screening program. With an overall major morbidity and mortality rate of 11.8%, it seems valid to discuss whether an endoscopic excision with advanced techniques with or without laparoscopic assistance would be preferable in this patient group, accepting a 6.6% reoperation rate for additional oncological resection with lymph node sampling in patients in whom a malignancy is found on histological analysis of the complete polyp.

## Introduction

In January 2014, a national bowel cancer screening program started in the Netherlands. This program is for men and women in the age group 55–75 years, and it is being implemented in phases between 2014 and 2019. The population screening is performed by immunological fecal occult blood testing, with subsequent colonoscopy if tests results are positive [1]. During colonoscopy, polyps are removed and biopsies are taken from lesions in the colon. Since polyps may progress to cancer over a period of time, this contributes to the prevention and early detection of colorectal cancer [2, 3]. From the bowel screening program in England, we know that cancer and higher risk adenomas were found in 11.6 and 43% of men and 7.8 and 29% of women, respectively [4].

The current treatment for patients with endoscopically unresectable polyps is to perform an oncological colorectal resection. However, a more radical mesenteric resection is generally associated with increased morbidity and mortality compared to a smaller resection, and these complications should therefore be weighed against the chance of preventing cancer [5]. Brigic et al. [6] remarked that clinical outcomes following oncologic resection for benign colonic polyps are poorly documented. Their prospective case controlled study showed similar postoperative complication rates after colonic resections for benign and malignant colonic polyps (46 and 31%, respectively).

Due to the introduction of the national screening program, an increase in patients referred for a segmental colectomy for benign, but endoscopically unresectable polyps, is expected. The aim of this study was to assess the impact on daily clinical practice of this increase and to compare pathological results to clinical outcome of resections.

## Materials and methods

This study is a retrospective cohort study from a prospectively maintained database. Medical records of consecutive patients, who underwent an oncological colorectal resection between January 2009 and December 2014 in our institution, were reviewed. Patients with polyps referred for surgery without microscopically proven cancer were identified. Exclusion criteria were macroscopic features of malignant degeneration noticed by the endoscopist during colonoscopy, masses that could not be passed with the endoscope and patients undergoing laparoscopic local excision.

Patient characteristics were assembled, including age, gender, body mass index (BMI), American Society of Anesthesiologists (ASA) classification and comorbidities. Information on preoperative and postoperative polyp pathology, type of surgery, postoperative complications and mortality were analyzed.

Resectability of a polyp was assessed by the endoscopist (expert opinion). Biopsies were taken during endoscopy in every patient. All patients were discussed in the weekly multidisciplinary gastrointestinal oncology meeting. All colorectal resections were performed laparoscopically by, or under supervision of, a specialized colorectal surgeon. Postoperatively, patients were managed according to the enhanced recovery after surgery (ERAS) fast track protocol [7]. Complications were graded according to the Clavien–Dindo classification [8], and major complications were defined as grade 3b or higher. Histological examination of the resected specimen and lymph node assessment were performed by pathologists according to a standardized protocol.

### Statistical analysis

Statistical analysis was performed using SPSS software, version 22.0 (SPSS Inc, Chicago, IL, USA).

Continuous variables were presented as mean values with a standard deviation (SD) or as median values with an interquartile range (IQR) according to the distribution. Discrete variables were presented as counts and percentages. Categorical data were compared between groups using the Chi-square test, and continuous data were compared using the independent samples *t* test or Mann–Whitney *U* test. A two-tailed *p* value of < 0.05 was considered statistically significant.

## Results

### Patient demographics

A total of 674 patients had an oncological colorectal resection between January 2009 and December 2014. Seventy-six patients met the inclusion criteria and were analyzed. Patients were predominantly male (56.5%) with a mean age of 65 years (range 38–82, SD 9.8). In 9 patients (11.8%), an invasive carcinoma was found after histological analysis of the resected specimen. Preoperative clinicopathologic characteristics of patients and polyps are summarized in Table 1.

**Table 1:** Characteristics of patients and polyps

Demographic	Total *N* = 76 (%*)	Benign *N* = 67 (%*)	Malignant *N* = 9 (%*)	*p* value
Age (years)				.739**
Median	65 (9.8)	66	63	
Range	38–82	38–82	39–72	
Sex				.723***
Female	33 (43.4)	30 (44.8)	3 (33.3)	
Male	43 (56.6)	37 (55.2)	6 (66.7)	
BMI				.931**
Average (SD)	27.08 (4.4)	27.03 (4.3)	27.44 (4.2)	
Range	17.94–38.20	17.94–38.20	22.82–35.16	
ASA classification				.757***
ASA 1	25 (32.9)	22 (32.8)	3 (33.3)	
ASA 2	42 (55.3)	36 (53.7)	6 (66.7)	
ASA 3	7 (9.2)	7 (10.4)	–	
ASA 4	1 (1.3)	1 (1.5)	–	
Type of operation				.009***
Right colectomy	45 (59.2)	44 (65.7)	1 (11.1)	
Left colectomy	8 (10.5)	4 (6.0)	4 (44.4)	
Sigmoidectomy/LAR	19 (25.0)	16 (23.9)	3 (33.3)	
Total colectomy	2 (2.6)	1 (1.5)	1 (11.1)	
Ileocaecal resection	2 (2.6)	2 (3.0)	–	
Preoperative histology				.024***
Tubulovillous	47 (62.3)	44 (65.7)	3 (33.3)	
Tubular	10 (13.0)	10 (14.9)	–	
Villous	7 (9.1)	4 (6.0)	3 (33.3)	
Other	10 (13.0)	7 (10.4)	3 (33.3)	
Unknown	2 (2.6)	2 (3.0)	–	
Dysplasia				.624***
Low grade	33 (43.4)	30 (44.8)	3 (33.3)	
High grade	13 (17.1)	11 (16.4)	2 (22.2)	
Unknown	30 (39.3)	26 (38.8)	4 (44.4)	
Total	76 (100)	67 (100)	9 (100)	

*BMI* body mass index, *ASA* American Society of anesthesiologists, *LAR* low anterior resection

* Unless otherwise stated in the first column

** Mann–Whitney *U* test

*** Chi-square test

### Outcome

Forty-seven of the 76 patients (61.8%) were operated on before the start of the screening program in 2014, and 29 patients (38.2%) after it had begun. All resections were planned as laparoscopic procedures, with a conversion rate of 3.9% (*n* = 3). A (nonsignificant) decrease in the percentage of malignant polyps was seen in patients treated in 2014 compared to the pre-screening cohort (7/47 = 14.9 vs. 2/29 = 6.9%, respectively) (*p* = .469).

Malignant polyps were found significantly more often after left sided colectomies versus other resections (44.4 vs. 10.4%, respectively, *p* < .001), whereas there was no significant difference in preoperative histological characteristics.

Although numbers were too small to compare tumor stage before and after the introduction of the program, it is noteworthy that all but 1 patient had early carcinoma without lymph nodes metastases (Table 2).

**Table 2:** Postoperative characteristics of invasive cancer

	*N* = 9 (%)
T stage	
T1	5 (55.6)
T2	3 (33.3)
T3	1 (11.1)
N stage	
N0	8 (88.9)
N1	1 (11.1)
Total	9 (100)

### Complications

The overall mortality rate was 2.6% (*n* = 2). Major complications (Clavien–Dindo > 3b) occurred in 9 patients (11.8%), with an anastomotic leakage rate of 3.9% (*n* = 3). Minor complications (Clavien–Dindo < 3b) occurred in 21.0% (*n* = 15) of patients (Table 3).

**Table 3:** Perioperative complications

Complications	*N* (%)
Perioperative death	2 (2.6)
Complication Clavien–Dindo > 3b	9 (11.8)
Complication Clavien–Dindo < 3b	16 (21.0)
Anastomotic leak	3 (3.9)
Conversion	3 (3.9)
Total	76 (100)

## Discussion

The number of patients referred for laparoscopic colorectal resection for benign polyps almost tripled after introduction of the national screening program in our country; 29 patients were operated on in 1 year (2014) compared to 47 patients in 5 years (2009–2013). Since the program is being introduced in phases, the number of patients referred for surgery after colonoscopy will most likely increase.

The malignancy rate dropped from 14.9 to 6.9% (nonsignificant difference), and apart from location (left sided), there were no predictive parameters for malignancy. Previous studies also showed a decrease in malignancy rate in polyps detected in a screening program. Patients from the UK bowel cancer screening program were more likely to have larger adenomatous polyps compared to the symptomatic population [4, 9, 10]. The underlying reasons for the differing profiles of polyps remain speculative, but this could also be an explanation for our decrease in the malignancy rate.

Multiple studies have shown that in the past endoscopists were not always experienced enough in differentiating benign from potentially malignant polyps. However, in recent years more training and the use of polyp classifications (e.g., Kudo pattern [11]) have increased their knowledge of the characteristics of a malignant polyp. The decline of the percentage of malignant polyps might also be explained by the improvement in this preoperative differentiation and the ongoing improvement in endoscopists’ ability to recognize malignant polyps.

Endoscopic submucosal dissection (ESD) is an advanced endoscopic technique that allows en bloc resection of gastrointestinal tumors [12], and implementing training of European endoscopists in ESD could have an important role in this setting.

Previous studies analyzing the incidence of malignancies in endoscopically unresectable polyps consequently recommend laparoscopic oncological resection and report malignancy rates in polyps varying from 8 to 22% [13–20] (Table 4). The morbidity and mortality reported in these studies were often poorly defined or not mentioned. The complication rate in the present study is consistent with the complication rates found in the total population undergoing colorectal surgery in the Netherlands [21]. In the largest study on unresectable polyps, Bertelson et al. [13] suggest that polyp size and villous features do not strongly predict malignancy, which seems consistent with the results of our study. They confirm that polyps located in the left colon are more likely to harbor cancer. Based on their findings, they suggest that endoscopically unresectable polyps are best treated by radical oncologic resection, but they do not describe morbidity or mortality in their population.

**Table 4:** Previous studies reporting the incidence of malignancy in endoscopically unresectable polyps thought to be being before resection with associated complication rates

Author	*N* patients	Malignancy rate (%)	Complications	Anastomotic leak	Mortality
Bertelson et al. [13]	750	17	Not mentioned	Not mentioned	Not mentioned
Loungnarath et al. [14]	165	13	23%	2.6%	2.6%
Hauenschild et al. [15]	58	0	9.3%	Not mentioned	0%
Itah et al. [16]	64	14	4%	1.7%	0%
Benedix et al. [17]	525	18	20.8%	3.6%	0.9%
Zmora et al. [18]	38	18	10.5%	2%	0%
Brozovich et al. [19]	63	22	Not mentioned	Not mentioned	Not mentioned
Adler et al. [20]	79	16	37%	Not mentioned	3%

As mentioned in the introduction, Brigic et al. [6] compared outcomes following segmental colectomy for benign and malignant colonic polyps. The fact that colectomy for benign disease was shown to cause significant morbidity in at least one-third of patients in their study should in their opinion provide further impetus to develop alternative, less invasive treatment options for this growing group of patients.

Particularly after open surgery, it is very undesirable to reoperate a patient to perform a completion lymphadenectomy after limited surgery for a presumed benign polyp.

Currently, given the laparoscopic skills of surgeons and improving recognitions of benign neoplasms of endoscopists a less invasive, laparoscopic-endoscopic rendezvous procedure could be a more favorable approach. In this procedure, the colonic segment containing the polyp can be laparoscopically manipulated to achieve better intraluminal exposure of the polyp. The endoscopic removal is with strict laparoscopic visualization of the serosal aspect of the polypectomy area, which allows any subtle change to be recognized and repaired if needed. For lesions inaccessible for endoscopic removal, a small colectomy for resection of the polyp or an endoscopy-assisted wedge resection could be performed [22–24]. It has to be determined whether these more limited procedures are associated with lower morbidity and mortality rates, shortened length of hospital stay and the advantage of preservation of the colon.

In this laparoscopic era, a completion lymphadenectomy within 2 weeks seems very feasible. If laparoscopic local excision would be an option rather than an oncological resection, only 6.6% of patients would need a reoperation for lymph node sampling and 93.4% would be spared a partial oncological colectomy.

Obviously the present study is limited by its retrospective design and relatively small numbers. Another limitation is the partially subjective nature of determining whether a polyp is endoscopically unresectable. However, all cases were discussed before surgery at a weekly multidisciplinary gastrointestinal oncology meeting.

The strength of the study is that it is a consecutive series from a single, non-academic, non-referral center, reflecting daily clinical practice and the correlation of pathological findings to clinical outcome. To our knowledge, this is the first study to report the increase in patients with benign polyps referred for surgery after implementation of a national bowel screening program.
